# Endodontic Retreatment of Maxillary Second Molar with Four Roots

**DOI:** 10.1155/2019/5348048

**Published:** 2019-05-21

**Authors:** Gabriel Magnucki

**Affiliations:** Zahnzentrum Schomaker & Magnucki, Bahnhofstrasse 16-18, 27211 Bassum, Germany

## Abstract

The present communication describes the endodontic retreatment of a rare four-rooted maxillary second molar. A 25-year-old patient was referred to our dental practice requesting an apicoectomy because of continuous and permanent pain reaction six months after the first endodontic treatment. The sent radiograph demonstrated three filled root canals (one mesial, two distal) and four radiographically superimposing roots (two mesial, two distal). Due to the diagnosed chronic apical periodontitis and based on the visible untreated root canal, we decided to endodontically re-treat the quadrangular tooth against the referred apicoectomy. In the first session, only the previously untreated mesiopalatal root canal was mechanically prepared and filled with a corticosteroid- and tetracycline-containing paste. After two symptom-free weeks, the gutta-percha was removed from the other canals and calcium hydroxide was applied. Another two weeks later, the four root canals, whose orifices were “irregular quadrilateral” shaped on the pulp chamber floor representing Versiani Type A, were obturated. After the verification of this rare anatomy by cone beam computed tomography, the tooth was classified as Christie's radicular type II. One characteristic of this type is parallel running buccal and palatal roots, which caused a radiographic superimposition and probably led to the endodontic mistreatment in the first case.

## 1. Introduction

Clear understanding of dental anatomy including root number and canal morphology is an essential prerequisite in endodontic therapies. In particular, anticipation of rare anatomical variations should be crucial for endodontic specialists. Such a dental anomaly is the presence of two palatal roots in human maxillary second molars. Libfeld and Rotstein described in 1200 investigated teeth the frequency of a four-rooted maxillary second molar at 0.4% [1]. Peikoff et al. identified in a retrospective study, containing 520 maxillary second molars with completed endodontic treatments, that 1.4% of the canadian patients had two palatal roots [2]. These results were recently confirmed by Martins et al. in 2018, who reported a similar occurrence rate in the Portuguese population using cone beam computed tomography [3]. Despite this rare incidence, publications concerning tooth anatomy describe several classifications for four-rooted maxillary molars [4–7]. The first characterization was indicated by Christie et al., who characterized maxillary molars with four roots (commonly two buccal and palatal) according to their shape and degree of separation of the palatal roots in three subtypes [4]. Baratto-Filho et al. suggested an additional class IV by demonstrating an endodontic case of fused mesiobuccal and mesiopalatal roots [8]. Furthermore, Versiani et al. indicated that Christie's classification is not feasible while studying four-rooted second maxillary molars [5]. They introduced a new type III, which is based on less divergent and short palatal roots along with widely divergent buccal roots by combining Christie's types II and III [4, 5]. Nevertheless, another classification exists for this rare anatomical anomaly conceived by Carlsen and Alexandersen (2000). They defined the additional palatal root as radix mesio- or distolingualis according to its direct affinity to the pronounced part of the crown [6]. Furthermore, they identified one case exhibiting three buccal roots in a maxillary first molar and classified it as radix paramolaris [7]. However, not all dental professionals are aware of this anomaly resulting in failed endodontic treatments. Therefore, the following case report describes an endodontic retreatment of a maxillary second molar with two palatal roots.

## 2. Case Report

**Figure 1:**
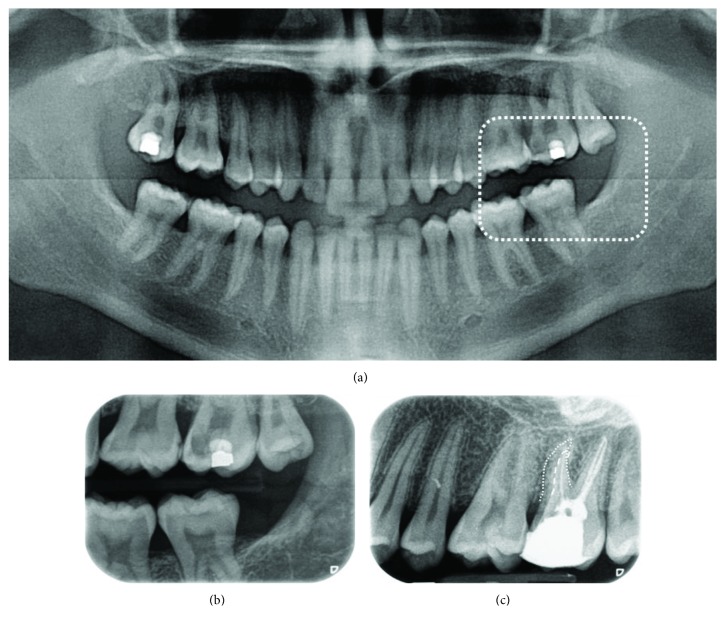
(a) Panoramic radiograph of the referred patient. (b) Bitewing view of the interesting tooth, which showed a deep carious lesion. (c) X-ray after the first endodontic treatment. In the distal roots, two root fillings are visible, whereas only one filled root canal can be identified in the mesial roots. Note two mesial roots. Buccal: dotted line; palatal: scored line.

**Figure 2:**
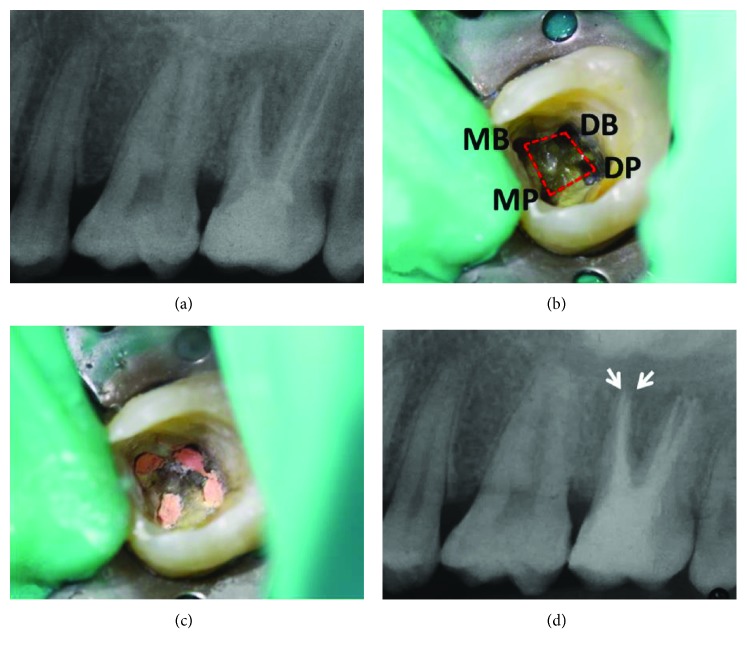
(a) The pretreatment radiograph showed an apical radiolucency on the mesial root. Due to the buccal-palatal superimposition, the second mesial root is not visible. (b) Photograph of the pulp chamber floor after removal of the gutta-percha. Root canal orifices were “irregular quadrilateral” shaped on the pulp chamber floor representing Versiani Type A. (c) Photograph after the canal obturation. (d) The posttreatment radiograph showed two mesial filled root canals (arrows).

A 25-year-old male patient was referred to our Department in Bassum, Lower Saxony, Germany, to receive apicoectomy on the maxillary left second molar. Together with the referral, the radiographic history of the relevant tooth was provided (Figures 1(a)–1(c)). The patient, whose medical history was noncontributory, complained of persistent pain reactions and tenderness of this tooth after a first endodontic treatment six months ago. Clinical examination showed a sufficient composite restoration, a painful tenderness to percussion, and no reaction to cold test. Tooth mobility and periodontal pockets were inconspicuous. No submucosal swelling was observed. The pretreatment radiograph showed an apical radiolucency on the mesial root and three radiopaque root fillings (two distal and one mesial), which led to the diagnosis of a chronic apical periodontitis. This straight radiographic evaluation of the examined tooth revealed no unusual anatomy (Figure 2(a)). But the X-ray image after the first endodontic treatment was taken mesial angulated, and here, a second previously untreated mesial root is visible (Figure 1(c)). Therefore, instead of the proposed apicoectomy, the decision was made to re-treat the root canals.

**Figure 3:**
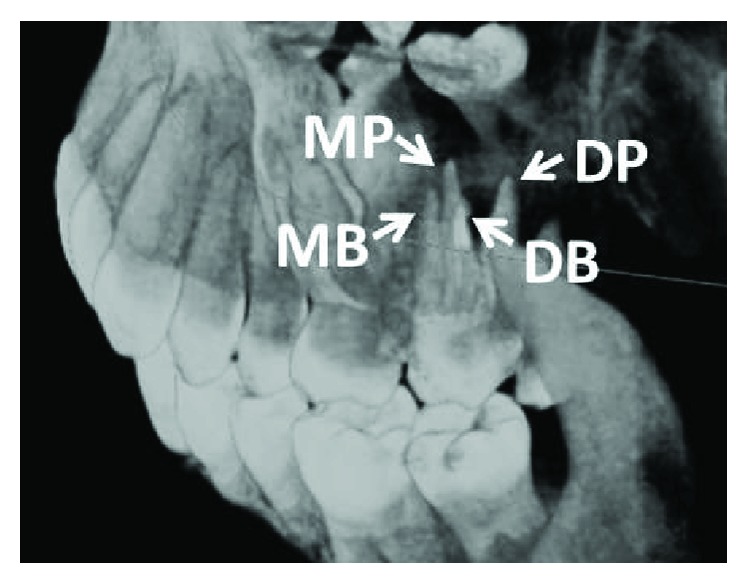
Cone beam computed tomographic 3D reconstruction of the treated tooth from a disto-buccal-apico angulation verifies the rare anatomy of four roots. MB: mesiobuccal; DB: distobuccal; MP: mesiopalatal; DP: distopalatal.

After the injection of 1 ml local anesthesia containing 40 mg articaine hydrochloride and 0.005 mg epinephrine (Septanest, Septodont, Saint-Maur-des-Fossés, France) and isolation with a rubber dam, the occlusal filling was removed. During the access preparation, the second (mesial), untreated palatal root canal orifices were found. Due to issues in the time management in the first treatment session, only the previously untreated root canal was explored with a manual instrument ISO 15, irrigated with 2.5% NaOCl, and corticosteroid- and tetracycline-containing paste (Ledermix®, RIEMSER Pharma, Greifswald, Germany) was applied with a lentulo spiral filler (ISO 25, Dentsply DeTrey, Konstanz, Germany). The access cavity was filled with Cavit (ESPE, Seefeld, Germany). Two weeks later, the patient described a symptom-free and painless period since the last treatment. During the second session, the gutta-percha in the coronal third of the root canals was removed with a diamond-coated ultrasonic tip (Komet Dental, Lemgo, Germany) and eucalyptus oil (Apotheke Gesundheitszentrum, Bassum, Germany) was applied for ten minutes. Afterwards, the gutta-percha was removed from the three filled canals (mesiobuccal, distobuccal, and distopalatal), and the canals were cleaned as well as shaped with a reciprocating instrument (R25, Reciproc®, VDW, Munich, Germany). The files were cleaned from the gutta-percha debris every three reciprocating units (“pecks”) according to the manufacturer's protocol. After the working length was reached (19 mm each canal (apex to the corresponding tooth cusps)), which was determined using an electronic apex locator (VDW.GOLD® RECIPROC®, VDW, Munich, Germany), the residual obturation material was removed ultrasonically (REDO setting, VDW.ULTRA®, VDW, Munich, Germany). The previously untreated canal (mesiopalatal) was also cleaned and shaped with a reciprocating instrument (R25, Reciproc®, VDW, Munich, Germany). Then, the root canals and especially the apical region were enlarged (R40, Reciproc®, VDW, Munich, Germany). During the instrumentation process, the canals were irrigated with 2.5% NaOCl, 2% CHX, and 18% EDTA and the liquids were activated ultrasonically (IRRI setting, VDW.ULTRA®, VDW, Munich, Germany). Afterwards, the disinfection was carried out with Ca(OH)_2_ (Calcicur®, VOCO, Cuxhaven, Germany), and the coronal seal was performed with Cavit (3M ESPE, Seefeld, Germany). Two weeks later, the canals were reentered and Ca(OH)_2_ was removed by using 2.5% NaOCl, 2% CHX, and 18% EDTA and obturated with appropriate gutta-percha mastercones (ISO 40, VDW, Munich, Germany) along with an AH plus sealer (Dentsply DeTrey, Konstanz, Germany) using the lateral condensation technique (Figure 2(c)). The permanent restoration was carried out using Tetric EvoCeram (Ivoclar Vivadent, Schaan, Liechtenstein). The postendodontic radiograph demonstrated two mesial-located apices (Figure 2(d)). To verify this rare anatomy, a 3D cone beam computed tomography (CBCT) (KaVo OP 3D DVT, KaVo Dental, Biberach an der Ri*β*, Germany) was performed and analyzed (Figure 3). The patient gave his informed consent for the publication of this communication.

## 3. Discussion

The incidence in the literature of four-rooted maxillary second molars varies between 0.22% in Thailand (*n* (number of investigated teeth) = 457) [9] and 2.4% in France (*n* = 167) [10]. Interestingly, also in other European populations, higher occurrence rates for two palatal roots in maxillary second molars were found in CBCT studies. In Italy, the frequency of upper second molars with four roots was described with 1.27% (*n* = 157) [11]. Comparable results were demonstrated in Greece (1.24%, *n* = 402) [12], in Cyprus (1.37%, *n* = 438) [13], and in the Portuguese study mentioned above (1.44%, *n* = 277) [3]. On the other hand, in East Asia, lower occurrence rates of this rare morphological anomaly were identified in population-based CBCT studies in Thailand (0.22%, *n* = 457) [9], South Korea (0.49%, *n* = 820) [14], and China (0.28%, *n* = 1066 [15]; 0.77%, *n* = 519 [16]; 0.98%, *n* = 1226 [17]; and 1.12%, *n* = 979 [18]). To clarify the root and canal anatomy of maxillary second molars and especially the global distribution of quadrangular teeth, a meta-analytical literature review would be supportive for the dental community. However, every dental professional and especially endodontists should be aware of the anatomical anomaly of a second palatal root to avoid treatment failures like the one reported in the present case. The rarity of this morphological aberration leads to the expectation of one case every three years in full-time endodontic practice [4]. For example, last year, we extracted a four-rooted maxillary first molar and analyzed its external and internal anatomy in our dental office [19]. After studying this anomaly intensively, we were able to interpret the sent radiographic images correctly. Hence, we decided for an endodontic retreatment against the proposed apicoectomy. Importantly, prior to an endodontic retreatment, all available information and especially radiographic examinations of the first root canal treatment should be obtained. In the present communication, four roots are only visible on the sent X-ray image of the referring colleague.

In clinical case documentations, Christie's classification is commonly used for the description of quadrangular teeth [4]. Christie et al. classified four-rooted maxillary molars according to shape, degree, fusion, and separation of the palatal roots [4]. Due to four separated roots and the parallel running morphology of the buccal and palatal roots, the presented tooth was identified as type II. A further characteristic for type II teeth is shorter roots, which explains the reduced working length in this case report. Remarkably, radiographic superimposition of the buccal and lingual roots in this type can lead to clinical misinterpretation such as in the present case [4]. In contrast, Christie's type I shows widely divergent, long, and tortuous palatal roots and the “cow horn”-shaped buccal roots demonstrate mainly four separate apices on radiographs [4]. Therefore, the diagnosis of this type is more feasible for experienced endodontists. Type III maxillary molars are “constricted in root morphology with the MB, MP and DP canals encaged in a web of root dentin.” In radiographs of this type, the DB root appears to stand alone [4]. In summary, the radiographic identification of quadrangular type II molars could be challenging and could complicate a correct diagnosis. However, in diagnostically difficult posterior regions, additional angulated radiographs could help the dentist to determine four-rooted molars. Nowadays, the endodontic usage of cone beam computed tomography can support the diagnosis of root and canal aberrations (Figure 3) [20].

After the correct diagnosis, the preparation of the trepanation cavity is the next endodontic challenge. Versiani pointed out that the root canal orifices were located mainly “irregular quadrilateral” (as in the present case) or “trapezoid” shaped on the pulpal chamber floor [5]. Therefore, the trepanation cavities have to be enlarged from the generally used triangular shape to trapezoid or quadrilateral for the localization of all root canal orifices [5].

## 4. Conclusion

Every dental practitioner and especially endodontists should possess deep knowledge of all existing anatomical variants and their radiographic characteristics to avoid treatment failures. Before the beginning of an endodontic retreatment, all available information of the first root canal treatment and the radiographic history of the tooth of interest should be obtained for the identification of possible radicular abnormalities.

## References

[1] Libfeld H., Rotstein I. (1989). Incidence of four-rooted maxillary second molars: literature review and radiographic survey of 1,200 teeth. *Journal of Endodontics*.

[2] Peikoff M. D., Christie W. H., Fogel H. M. (1996). The maxillary second molar: variations in the number of roots and canals. *International Endodontic Journal*.

[3] Martins J. N. R., Marques D., Francisco H., Caramês J. (2018). Gender influence on the number of roots and root canal system configuration in human permanent teeth of a Portuguese subpopulation. *Quintessence International*.

[4] Christie W. H., Peikoff M. D., Fogel H. M. (1991). Maxillary molars with two palatal roots: a retrospective clinical study. *Journal of Endodontics*.

[5] Versiani M. A., Pécora J. D., de Sousa-Neto M. D. (2012). Root and root canal morphology of four-rooted maxillary second molars: a micro-computed tomography study. *Journal of Endodontics*.

[6] Carlsen O., Alexandersen V. (2000). Radix mesiolingualis and radix distolingualis in a collection of permanent maxillary molars. *Acta Odontologica Scandinavica*.

[7] Carlsen O., Alexandersen V. (1999). Radix paramolaris and radix distomolaris in Danish permanent maxillary molars. *Acta Odontologica Scandinavica*.

[8] Baratto-Filho F., Fariniuk L. F., Ferreira E. L., Pecora J. D., Cruz-Filho A. M., Sousa-Neto M. D. (2002). Clinical and macroscopic study of maxillary molars with two palatal roots. *International Endodontic Journal*.

[9] Ratanajirasut R., Panichuttra A., Panmekiate S. (2018). A cone-beam computed tomographic study of root and canal morphology of maxillary first and second permanent molars in a Thai population. *Journal of Endodontics*.

[10] Monsarrat P., Arcaute B., Peters O. A. (2016). Interrelationships in the variability of root canal anatomy among the permanent teeth: a full-mouth approach by cone-beam CT. *PLoS One*.

[11] Plotino G., Tocci L., Grande N. M. (2013). Symmetry of root and root canal morphology of maxillary and mandibular molars in a white population: a cone-beam computed tomography study *in vivo*. *Journal of Endodontics*.

[12] Nikoloudaki G. E., Kontogiannis T. G., Kerezoudis N. P. (2015). Evaluation of the root and canal morphology of maxillary permanent molars and the incidence of the second mesiobuccal root canal in Greek population using cone-beam computed tomography. *The Open Dentistry Journal*.

[13] Kalender A., Celikten B., Tufenkci P. (2016). Cone beam computed tomography evaluation of maxillary molar root canal morphology in a Turkish Cypriot population. *Biotechnology & Biotechnological Equipment*.

[14] Kim Y., Lee S. J., Woo J. (2012). Morphology of maxillary first and second molars analyzed by cone-beam computed tomography in a Korean population: variations in the number of roots and canals and the incidence of fusion. *Journal of Endodontics*.

[15] Wang H., Ci B. W., Yu H. Y. (2017). Evaluation of root and canal morphology of maxillary molars in a Southern Chinese subpopulation: a cone-beam computed tomographic study. *International Journal of Clinical and Experimental Medicine*.

[16] Jing Y. N., Ye X., Liu D. G., Zhang Z. Y., Ma X. C. (2014). Cone-beam computed tomography was used for study of root and canal morphology of maxillary first and second molars. *Beijing Da Xue Xue Bao Yi Xue Ban*.

[17] Gu Y., Wang W., Ni L. (2015). Four-rooted permanent maxillary first and second molars in a northwestern Chinese population. *Archives of Oral Biology*.

[18] Yang B., Lu Q., Bai Q. X., Zhang Y., Liu X. J., Liu Z. J. (2013). Evaluation of the prevalence of the maxillary molars with two palatal roots by cone-beam CT. *Zhonghua kou Qiang yi xue za zhi*.

[19] Magnucki G., Kahl W. A. (2018). Four rooted maxillary first molar with five root canals and two enamel pearls. *International Journal of Dental and Health Sciences*.

[20] Venskutonis T., Plotino G., Juodzbalys G., Mickevičienė L. (2014). The importance of cone-beam computed tomography in the management of endodontic problems: a review of the literature. *Journal of Endodontics*.

